# Relaxation of the Utero-Sacral Ligaments—An Outline Intended Only to Introduce the Subject for Discussion in Atlanta Society of Medicine March 4th, 1890*This paper was not intended for publication, therefore this fact is offered as an apology for its lack of preparation.

**Published:** 1890-04

**Authors:** Geo. H. Noble

**Affiliations:** Atlanta, Ga.


### RELAXATION OF THE UTERO-SACRAL LIGAMENTS —AN OUTLINE INTENDED ONLY TO INTRODUCE THE SUBJECT FOR DISCUSSION IN ATLANTA SOCIETY OF MEDICINE, MARCH 4TH, 1890.*

BY GEO. H. NOBLE, M. D., ATLANTA, GA.

The functions of the utero-sacral ligaments are to aid in the support of the uterus. They, together with the vesical attachments and the base of the bladder, form a sort of suspension bridge, with either extremity attached anterio-posteriorly to the bony pelvis. The uterus sits about the middle of this bridge, with the body considerably inclined forward, and the cervix directed backward, where it meets the axis of the vagina perpendicularly.

The ligaments consist, in great part, of folds of peritoneum, connective, cellular tissue, and some few muscular fibres. They diverge from the sides and rear of the uterus, and pass back by either side of the rectum to the sacrum, with Dungla's pouch dipping down between them, thus forming an elongated opening for the passage of the bowel from the abdominal cavity. This arrangement is a wise provision of nature against frequent strictures of the rectum, for if the intestine were to pass out from the peritoneal cavity through a circular opening just large enough to admit it (with the ligaments on either side,) inflammatory deposits would prove a frequent source of trouble by impinging upon the lumen of the bowel. As it is, all that space between the ligaments, extending from the rectum

* This paper was not intended for publication, therefore this fact is offered as an apology for its lack of preparation.

to the uterus, must be obliterated before an irreparable stricture can occur.

There is a natural tendency of the uterus to flexions and version forward. Of this we see frequent instances in the virgin. Normal or unfixed flexions bear evidence of the fact. It is gravitation acting upon the uterus, already leaning in that direction—in other words, the uterus tumbles down hill. In the opposite direction force is required to throw the uterus out of place so long as the parts are in a healthy condition. The position of the uterus must be so changed that the center of gravity is moved to a point in the rear of the cervix before gravitation alone can effect displacements backward. But with the ligaments in a state of relaxation (a thing so commonly associated with and following upon engorgement, subinvolution and other conditions entailing an increased weight of the uterus), we find the center of gravity gradually changing from the anterior to the rear of the cervix. The ligaments stretch from the super-imposed weight and altered circulation, the uterus drops down in the pelvis, the cervix follows the axis of the vagina, and nears the pubic arch, as it approaches the vaginal outlet. The swelled fundus follows the same curve, and is thrown backwards as the cervix swings forward until it finds its resting place in the hollow of the sacrum. If it progresses far enough, the weight from above and pressure from the abdominal muscles may force the fundus down between utero-sacral ligaments, which may then form a natural ligature, constricting the superficial veins, and pampiniform plexus to a greater or less extent, causing the venous stasis and swelling to become so great as to impact the uterus beneath the ligaments, and require some force to dislodge it. This I have demonstrated upon the living subject, and upon a woman who had been said to have incurable adhesion. I had long since noticed that admission of air to the vagina, with the patient in the genu-pectoral position, failed in a great many cases to replace the uterus. And in many instances considerable pressure was necessary to start the uterus, after which it was readily reducible. It seemed to me that it was held back by a vacuum force, and that as soon as it was disturbed the resistance was relieved. Adhesions were excluded, for the uterus would become fixed as often as it was permitted to become displaced, and as no symptoms indicative of rupture of lymph bands occurred.

The replacement was always more difficult when the utero-sacral ligaments were made tight by traction upon the cervix. Slight tension or movements with a hook in the cervix would sometimes aid me, but traction would always interfere.

When traction is made upon the ligaments, and force sufficient to press the uterus between them is used, the organ will go over with a very appreciable jump or start. Then, if the uterus be allowed to reassume its abnormal position, the above signs or features will again be demonstrated. But if the patient be placed upon the back, and the uterus lifted high up in the pelvis, it will be replaced without this resistance, and the sudden giving way of the same.

This can also be demonstrated with the patient upon the knees, if the uterus first be elevated in the pelvis with a view of relaxing the utero-sacral ligaments.

The adoption of one or more of the various means of relief will be governed by circumstances, the patient's surroundings and position in life. Our desire would be to restore, as far as possible, the natural condition of things; therefore, shortening of the ligaments, the supports from above would most naturally occur to us. But this is a dangerous and unsatisfactory process. If the stretching was confined to a portion of the ligaments only, the weak place might be remedied, but they are diseased through their whole extent, and therefore would require reinforcement in their entirety, which is impossible. If it were possible to double them upon themselves in such a way as to make a double or triple ligament, the union would be between two peritoneal surfaces, which would soon stretch away from the tissues underneath, and the result would be disappointing. Add to this the inaccessibility to the average operator of the utero-sacral ligaments, and we have but little encouragement for bold measures, unless it be in exceptional cases peculiarly suitable for such work. Even then I should not feel content to rest upon its accomplishment alone, but would supplement it by support from below, either in the form of a pessary or an operation designed to diminish the calibre of the vaginal canal, and to support the uterus and circulation below it. These latter, probably, are safer in the hands of the profession at large, though there are but very few persons that can perform a colporraphy posterior that will be of any material benefit to the patient. Certainly the drawing together of the mucous membrane is useless except for the time being.

At times it will be found impossible to reduce the size of the uterus until some surgical procedure has been adopted, such as closure of the cervix or its amputation when hypertrophical or perhaps a colporraphy I have had closure of cervical laceration and reposition of the uterus by pessaries fail to reduce the uterus in size, and then have the uterus in the same case reduced over fifty per cent. in two weeks from a successful colporraphy posterior. So replacement of the uterus, whether by shortening of the ligaments or otherwise, and closure of the cervix, does not always mean cure, especially if the patient is left with a large baggy vagina, and the pelvis below the uterus full of distended veins. I believe it is just as necessary to correct the perverted circulation in the lower pelvis as it is in the uterus or broad ligaments, for if you do not you not only fail to get support from below, but have a heavy weight of blood and tissue dragging the uterus down.

Now, as to what I regard as a successful colporraphy posterior, I mean to tell you next fall, and report at the same time a number of successful operations for prolapsus and complete procidentia. But before quitting this subject I will mention another operation that perhaps I might suggest for complete relaxation of the utero-sacral ligaments, or procidentia, and that is vaginal hysterectomy. This seems a wanton procedure as long as we have other means that will make our patient comfortable without the danger; but prominent men on both sides of the Atlantic are doing it for this cause. They are the most favorable class of cases for the operation, therefore serve a double purpose—one of which is to gratify the ambition of the surgeon.

---

*Error*—On page 154, tenth line from bottom, *Dungla's* should be *Douglas'*.

